# Circumcision of Women

**Published:** 1904-09

**Authors:** 


### Circumcision of Women.

[From *Caledonian Medical Journal*, July, 1904.]

THE inhabitants of Egypt are circumcised. Religion prescribes it to the Mahometans, and the Copts, or Catholics, willing to double their claims to paradise, are circumcised as well as baptised. Circumcision was practised among the ancient Egyptians, and some have gone so far as to find it necessary in the climate of that country.

In almost every other region, men alone are circumcised, and the mode of operation is universally known, but in Egypt women likewise submit to the operation, or to an operation somewhat similar, and one which has obtained the same name. The circumcision of women was likewise practised by the ancient Egyptians. It is at present confined to women who are properly Egyptians; the women of other countries, who are brought thither, having no occasion for the operation.

Sonnini justly remarks that it is by no means easy to treat of this subject with any degree of perspicuity, and, at the same time, to avoid such expressions as may offend the ear of modesty. The subject, however, is of importance in the history of man. To pass it over would be improper. It will be necessary, therefore, in discussing this subject to make use of the scientific terms which, though they may not be so universally understood, are certainly less offensive to delicacy.

That the women of Egypt undergo an operation denominated circumcision has long been known, but writers remain ignorant of the nature of the operation. Many affirmed that it consisted in retrenching the nymphæ, which were supposed to grow to an uncommon size. Bruce distinguished the operation by the name of excision, and affirmed that it consisted in shortening the clitoris. Niebuhr seems to have adopted an opinion compounded of these two notions, and such was the idea of Sonnini himself, till he had an opportunity of procuring more exact information.

That the nymphæ and clitoris are frequently retrenched seems to Sonnini, after the enquiries he made, to be sufficiently certain; but this is not all that is understood by the circumcision of women. Sonnini had long been of opinion that the ideas entertained of this operation were not correct. He wished to examine the matter more accurately, and he formed a project which, in Egypt, was not a little arduous—he resolved to witness the circumcision of a girl.

Forneti, the interpreter, lent his aid in this affair, which it was by no means easy to accomplish. The impropriety of exposing to the profane eyes of a Frank a Mahometan girl was strongly urged. Besides, it was winter, and deep-rooted prejudice had fixed the commencement of the rise of the Nile as the only auspicious period for the performance of the operation. The only argument in opposition to such objections was money, and this was so well applied that Sonnini at length had an opportunity, not only of seeing the operation performed, but also of examining a case which had been operated upon some years previously. When he examined her person, Sonnini perceived a fleshy excrescence hang from the os pubis, immediately above the labia. Its length was about half an inch, and the removal of this excrescence was what constituted female circumcision. The operator made the girl sit down on the ground before her, and, with a bad razor, immediately cut off the part mentioned. Though a good deal of blood issued from it, nothing was applied except a small quantity of ashes. Neither the nymphæ nor the clitoris were touched, and, indeed they were not visible in this girl or in her who had formerly been circumcised.

Circumcision in the women of Egypt, if this operation can be properly called by that name, is the effect rather of necessity than of choice. The excrescence is enlarged in proportion to the age of the woman, the operator assuring Sonnini that, if it is suffered to remain untouched, at the age of 25 it would be 4 inches in length. The operation is always performed, however, before the girls arrive at maturity, generally about the age of 7 or 8. The operators, who are, for the most part, women, are commonly natives of Upper Egypt. At the season reckoned most proper for the performance, which has already been mentioned to be the commencement of the increase of the Nile, they go from village to village proclaiming their occupation.

The excrescence which renders circumcision necessary is peculiar to women of Egyptian origin—to women descended from the ancient race of inhabitants. Females brought from another country, or females descended from parents who at some period, however remote, have arrived from another country, never exhibit any such excrescence. If we compare this circumstance with the accounts of the women in other parts of Africa, it may tend to render descriptions, formerly much suspected, if not wholly true, at least partly true. The preternatural apron of the Hottentot females was long considered as a fable, and Vaillant himself, who had traversed many parts of the country, strenuously contradicted the supposition of any such appearance. The same traveler, however, at a great distance from the Cape of Good Hope, met with women with a lengthened excrescence, similar to that belonging to the Egyptian women, except that at the lower extremity it was divided into two parts. The latter circumstance he considers as the effect of art.

Should the dubious authority of Vaillant be reckoned insufficient to establish the reality of any such conformation existing among the Hottentot females, his assertion may be confirmed by the authority of one whose veracity has not been suspected. Barron, in his travels near the Cape, mentions the same circumstance. It is evident, therefore, that at the two extremities of Africa, are found women who from nature have received such an excrescence.

Female circumcision, at the same time, is known to be practised in Abyssinia, and, though the nature of that circumcision has not been precisely determined, it may be presumed to be owing to a conformation similar to that of the Egyptian women, especially when we consider that those who perform circumçision in Egypt come from that part of the country which is nearest Abyssinia. Among the African negroes, no such conformation has ever been observed. Upon the whole, therefore, it may be concluded with some degree of probability, that the peculiarity in question is confined to the tawny women in Africa, and that a race of them extends, perhaps with a few interruptions, from Egypt in the north to the Cape of Good Hope in the south. It is probable, likewise, that it is peculiar to a race of women, without being under the influence of climate, since no length of residence in Egypt seems to bestow such a mark on females not belonging to the original inhabitants. It would be curious to know whether the original Egyptians preserve the same conformation after a residence of successive generations in a different country. Should this appear to be the case, it would afford a very powerful argument in favor of those who contend for the existence of different species of human beings.

